# Studies of Novel Coronavirus Disease 19 (COVID-19) Pandemic: A Global Analysis of Literature

**DOI:** 10.3390/ijerph17114095

**Published:** 2020-06-08

**Authors:** Bach Xuan Tran, Giang Hai Ha, Long Hoang Nguyen, Giang Thu Vu, Men Thi Hoang, Huong Thi Le, Carl A. Latkin, Cyrus S.H. Ho, Roger C.M. Ho

**Affiliations:** 1 Institute for Preventive Medicine and Public Health, Hanoi Medical University, Hanoi 100000, Vietnam; lethihuong@hmu.edu.vn; 2 Bloomberg School of Public Health, Johns Hopkins University, Baltimore, MD 21205, USA; carl.latkin@jhu.edu; 3 Institute for Global Health Innovations, Duy Tan University, Da Nang 550000, Vietnam; hahaigiang@duytan.edu.vn (G.H.H.); hoangthimen@duytan.edu.vn (M.T.H.); 4 Faculty of Pharmacy, Duy Tan University, Da Nang 550000, Vietnam; 5 VNU School of Medicine and Pharmacy, Vietnam National University, Hanoi 100000, Vietnam; nhlong.smp@vnu.edu.vn; 6 Center of Excellence in Evidence-based Medicine, Nguyen Tat Thanh University, Ho Chi Minh City 700000, Vietnam; giang.coentt@gmail.com; 7 Department of Psychological Medicine, National University Hospital, Singapore 119074, Singapore; cyrushosh@gmail.com; 8 Department of Psychological Medicine, Yong Loo Lin School of Medicine, National University of Singapore, Singapore 119228, Singapore; pcmrhcm@nus.edu.sg; 9 Institute for Health Innovation and Technology (iHealthtech), National University of Singapore, Singapore 117599, Singapore

**Keywords:** scientometrics, content analysis, text mining, COVID-19

## Abstract

Novel coronavirus disease 19 (COVID-19) is a global threat to millions of lives. Enormous efforts in knowledge production have been made in the last few months, requiring a comprehensive analysis to examine the research gaps and to help guide an agenda for further studies. This study aims to explore the current research foci and their country variations regarding levels of income and COVID-19 transmission features. This textual analysis of 5780 publications extracted from the Web of Science, Medline, and Scopus databases was performed to explore the current research foci and propose further research agenda. The Latent Dirichlet allocation was used for topic modeling. Regression analysis was conducted to examine country variations in the research foci. Results indicate that publications are mainly contributed by the United States, China, and European countries. Guidelines for emergency care and surgical, viral pathogenesis, and global responses in the COVID-19 pandemic are the most common topics. There is variation in the research approaches to mitigate COVID-19 problems in countries with different income and transmission levels. Findings highlighted the need for global research collaborations among high- and low/middle-income countries in the different stages of pandemic prevention and control.

## 1. Introduction

Novel coronavirus disease 19 (COVID-19), caused by severe acute respiratory syndrome coronavirus 2 (SARS-CoV-2), is currently threatening millions of lives in the world. Since the first introduction at the end of 2019, this disease was officially declared as a global pandemic by the World Health Organization (WHO) on March 11, 2020 [1]. Until April 30, 2020, 185 countries/territories reported 3.2 million confirmed cases with 227,847 total deaths [2], and the highest-burden has been placed in European and American countries [1]. Serious health, social, and economic consequences of COVID-19 have been well-recognized [3,4,5,6,7], especially among the elderly with comorbidities, homeless individuals, and also residents who face financial, mental, and physical hardships due to social distancing policies [8]. 

Given that COVID-19 is a new threat without any antiviral therapies or vaccines, current measures to mitigate this crisis depend heavily on the national and regional preparedness and responses [9]. However, optimal strategies to cope with the complexity of this pandemic demand substantial scientific evidence. Recently, the WHO has issued technical guidance for countries/regions and research institutions, as well as having worked closely with global researchers to update the empirical evidence [10,11]. Efforts have been made around the globe to enhance the understanding of the COVID-19’s dynamic transmission, develop effective vaccines and treatment regimes, as well as evaluating impacts of current responses on different populations’ health and well-being [12]. As a result, in the last four months, the number of COVID-19-related publications has increased dramatically in various forms including articles, reviews, letters to editors, or preprint documents [13]. These contributions have proven the importance of scientific research in pandemic preparedness and helped governments to respond rapidly and effectively to the crisis [14]. 

The current growth body of literature has rapidly shaped our knowledge about COVID-19, but it also raises the need to identify the remaining research questions that should be prioritized [15]. However, there has been a lack of studies attempting to identify the country and regional variations in COVID-19-related research foci. Several systematic reviews have been conducted to examine the clinical characteristics of COVID-19 [16,17], or the effectiveness of specific COVID-19 treatment and policies [18,19,20]. However, these studies only focused on a specific aspect of COVID-19, as well as only reviewing a small volume of articles, which are unable to capture a comprehensive picture of global COVID-19 research. One potential solution to address these limitations is bibliometric analysis. By using systematically quantitative analyses for a vast amount of publications, this method is widely used to quantify the growth of research productivity, the most prolific countries and institutions, and the development of research contents [21,22,23]. In this paper, we used the bibliometric analysis with aims to explore the current research foci and their country variations regarding levels of income and COVID-19 transmission features. Findings of this study would potentially inform current knowledge gaps about COVID-19, as well as propose future research directions.

## 2. Materials and Methods

### 2.1. Searching Strategy and Study Selection

**Figure 1:**
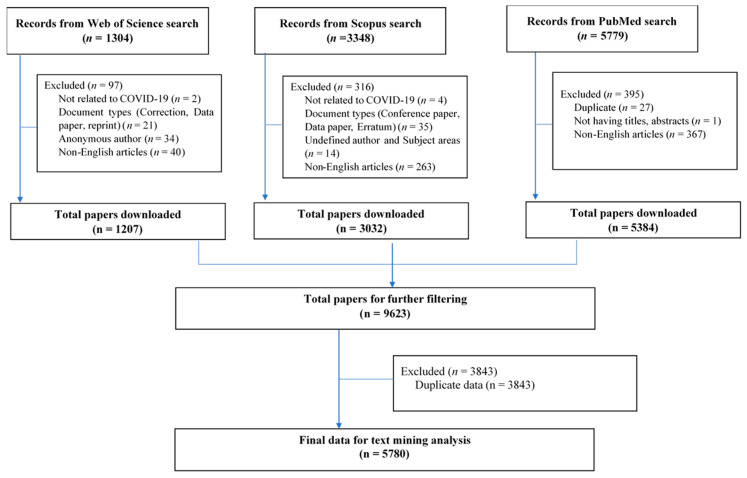
Selection process.

Information on COVID-19 and SARS-CoV-2-related documents published until 23 April 2020 were extracted from the Medline, Scopus, and Web of Science (WoS) databases. These databases allowed us to retrieve essential information for bibliometric analysis including title/abstract, keywords, number of citations, and authors’ affiliations, which might not be available in other databases (such as Embase or Science Direct). We did not use preprint databases (e.g., bioRxiv, arXiv, or medRxiv) for searching process since publications in these databases have not undergone the peer-reviewed process, which might hinder their quality. The search terms and search queries for each online database were developed according to the WHO naming process for the virus and the disease it causes [24], and are presented in Table A1, Table A2 and Table A3. Any English-language publications containing COVID-19 disease or SARS-CoV-2 virus published from December 2019 to 23 April 2020 were included. Document types such as corrections, data papers, reprints, or conference papers were excluded because they might be duplicated in peer-reviewed papers. Datasets of three databases were merged, and duplications were screened independently and removed by two researchers. A final dataset of 5780 papers was used for further analysis. The searching process was presented in Figure 1. 

### 2.2. Data Analysis

In this paper, we extracted data on documents’ title, abstract, keywords, citation, and authors’ affiliation for analysis. As a document could be authored by scholars from different countries, we considered that all these countries contributed to the document preparation. Moreover, we decided to include both documents with, and without, abstracts for text analysis since the title of the document could partly reflect the document’s topic. We first descriptively analyzed the number of publications in each country and presented these data by using Microsoft Excel’s Map function. Then, we exported the top ten most cited publications for a detailed analysis of these papers’ content.

We used the VOSviewer software (version 1.6.15, Centre for Science and Technology Studies, Leiden University, the Netherlands) to illustrate the networks of the co-occurrence of keywords and most frequent terms in title/abstract [25,26]. Then, we employed Latent Dirichlet allocation (LDA) to discover fifteen latent topics from the titles and abstracts of documents. This Bayesian model treats each document as a set of topics, and topics are probability distributed over a set of words and their co-occurrence [27]. Thus, the LDA technique can produce two outputs: (1) probability distributions of different topics per document (to acknowledge how many topics are created based on the given publications), and (2) probability distributions of unique words per topic (to define the topic) [27]. Because each title/abstract may contain a mixture of topics, the LDA outputs may not reflect a specific research field or discipline. However, experiences from previous work suggested that documents focusing on a particular theme would be more likely to be categorized in the same group. To assure the robustness in labeling each topic, we checked at least ten documents per topic to ensure that the theme’s name could generally fit the content of documents.

Multivariable linear regression models were performed to examine the research foci of countries with different income classifications (low, low-middle, high-middle, and high income—according to the World Bank classifications) [28], and different COVID-19 transmission classifications (Pending, Sporadic case, Clusters of cases, Community transmission—according to the WHO classifications) [29]. The dependent variable was the share of publications in specific topic out of total publications in each country (%), while the main independent variables were income classifications and transmission classifications. The models were adjusted to the natural logarithm of gross domestic product (GDP) per capita, the number of COVID-19 cases, and the number of COVID-19 deaths per country. The latest data on GDP per capita and income classifications were collected from the World Bank database, while data on COVID-19 cases and deaths were extracted from WHO reports on 24 April 2020. A p-value of less than 0.05 was used to detect statistical significance. 

## 3. Results

**Figure 2:**
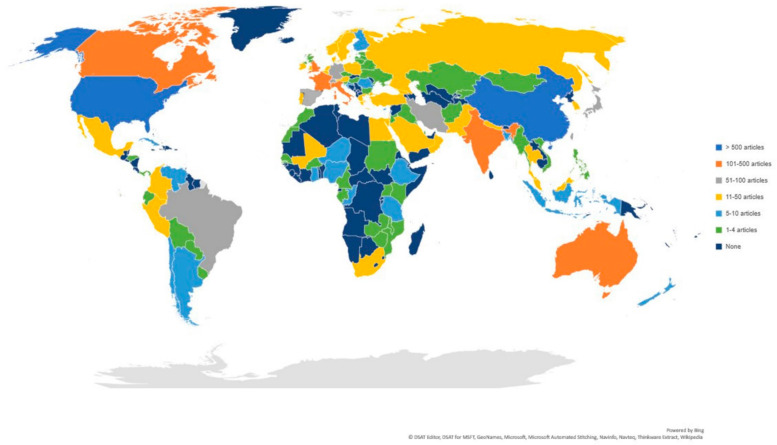
Number of publications per country

Figure 2 shows the research productivity of each country. A total of 115 countries produced 5780 publications in the searching period. It appears that scientific publications were mainly driven by the research hubs such as China, the United States, Canada, France, Italy, the United Kingdom, and India, which were also heavily hit by the COVID-19. In contrast, the majority of African countries had no more than 10 publications about COVID-19.

The list of ten most cited publications about SARS-CoV-2 and COVID-19 and their main findings are presented in Table 1. Reports on the clinical and laboratory characteristics of the confirmed cases are of the most interest, with six out of ten papers in the list. The most cited paper was a descriptive study about epidemiological and clinical features of 99 cases from Wuhan, China, which was believed to be the genesis of SARS-CoV-2. 

**Figure 3:**
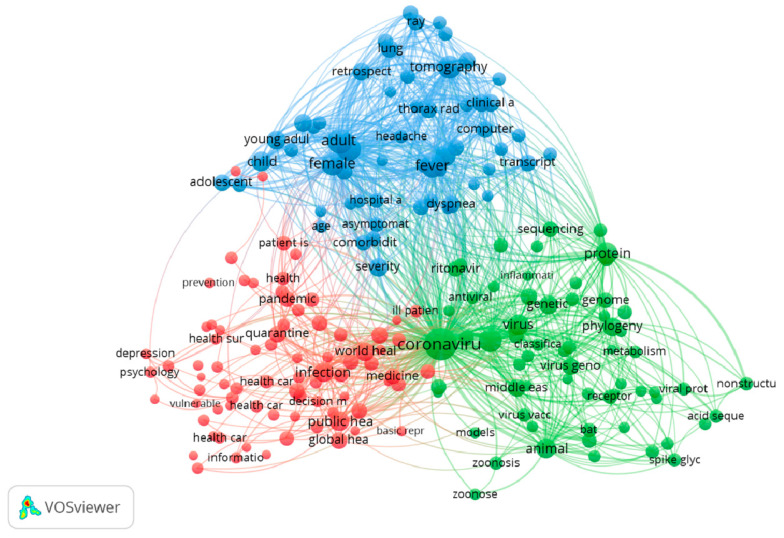
Co-occurrence analysis of keywords.

Figure 3 presents the network of 200 keywords with a co-occurrence of at least 20 times. The keywords were assigned to three major clusters. Cluster 1 (blue) reveals some basic imaging techniques for the diagnosis of lung function impairments (tomography and thorax radiograph) in children, adolescents, and adults. Cluster 2 (red) refers to the major concerns of the world regarding COVID-19, such as prevention, medicine, and public health response. Cluster 3 (green) focuses on the biology of SARS-CoV-2, including the origin, the phylogenetic network, and the genomic, proteomic, and metabolomic characteristics of the virus.

**Figure 4:**
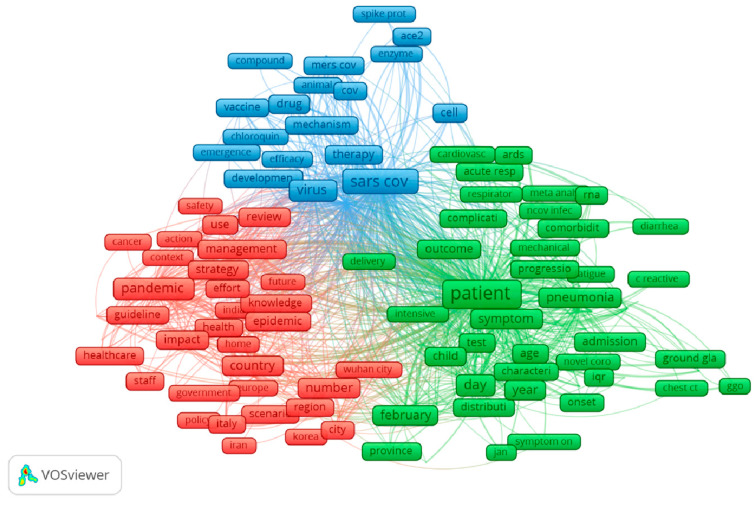
Co-occurrence analysis of the most frequent terms.

Thematic analysis of 250 most frequent terms is presented in Figure 4. Major themes of current research on COVID-19 are (1) promising therapies for COVID-19 prevention and treatment, and their mechanisms (blue); (2) hot spots of the pandemic and governments’ responses (red); and (3) clinical patterns and complications of COVID-19 (green). 

**Figure 5:**
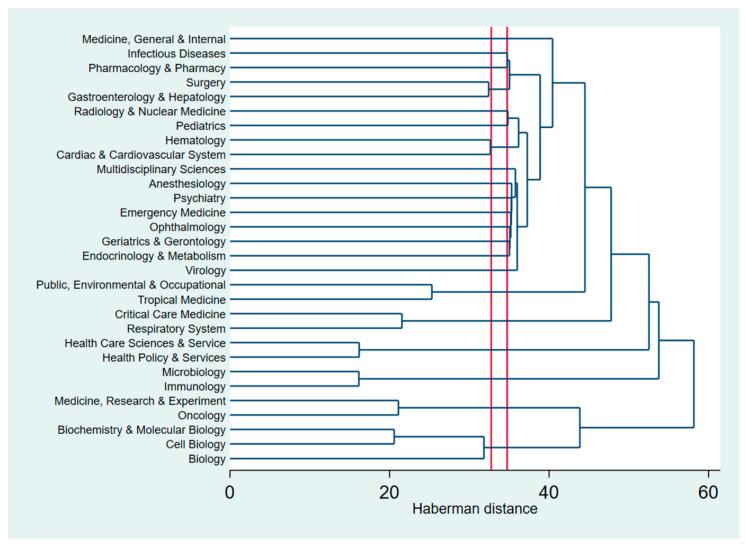
Dendrogram of research areas in WOS database.

Figure 5 shows the dendrogram analysis which indicates clustering of research areas in the WOS database. The research landscapes were the combination of several research areas. The first cluster was Infectious diseases and Pharmacology. This cluster has a close connection with Surgery and Gastroenterology (second cluster). The third cluster relates to treatment and diagnosis (such as Radiology, Hematology, Virology, Psychiatry, Gerontology, or Metabolism). The other clusters in COVID-19 research areas include (1) Critical care and Respiratory System (the fourth cluster), (2) Health care service and Health policy (the fifth cluster), (3) Microbiology and Immunology (the sixth cluster), (4) Oncology and Experimental Research (the seven cluster), and (5) Biology (the eight cluster).

The LDA results are presented in Table 2. Overall, researchers have devoted special attention to the biology of SARS-CoV-2 (Topics 3 and 4) and made an enormous effort on various aspects of clinical investigations, such as diagnostic tests for virus detection, clinical examination, and treatment for hospitalized patients (Topic 5, 7, 8, 9, 10, 11, and 15). Meanwhile, research on global and national responses to COVID-19 accounts for nearly a quarter of available publications (Topic 2, 12, and 13). Epidemiological characteristics of COVID-19 and psychological disorders during the epidemic are also of great interest (Topic 1, 6, and 14). 

The country variations in research foci are shown in Table 3. High-income countries (HICs) showed less attention on research in epidemiological characteristics and interventions of psychological disorders in the COVID-19 pandemic (Topic 6) compared with countries with other income levels. Meanwhile, low-middle income countries were found to have a less interest in diagnostic values of SARS-CoV-2 tests and improvement strategies (Topic 10) compared to low-income countries. Treatment interventions for COVID-19 (Topic 15) attracted the interest of scientists among countries at all income levels, especially in HICs. 

Regarding transmission classifications, comorbidities in patients with COVID-19 (Topic 8) were found to receive less attention among countries with sporadic cases in comparison with countries having “pending” transmission classification. Treatment interventions had less attention in countries having sporadic cases, a cluster of cases, and community transmission compared with those with “pending” transmission classification.

## 4. Discussion

By using LDA as the natural language processing approach, this study was able to capture the foci of COVID-19 related publications in different settings. This paper informed the rapid growth of research publications, and the global variation in research productivity and research interests. Moreover, findings of this study indicated that global scholars are paying attention to clinical management, viral pathogenesis, and public health responses, while other issues, such as psycho-social problems or impacts of COVID-19 on different vulnerable populations, are not-well investigated.

In this study, we found a greater number of publications regarding COVID-19 and SARS-CoV-2 in comparison with previous bibliometric studies [21,22,23]. For example, Lou et al. used the Medline database and only found 183 publications through February 29, 2020 [22]. This disparity could be justified that our search was far more comprehensive than these studies by using three major databases including the Medline, Scopus, and WOS. In addition, we included other document types such as letters, commentaries, or notes rather than concentrating only on original articles. As original papers require a long period for peer-review [30], scientists tended to publish their ideas in those document types first for receiving rapid feedbacks from others [31]. Therefore, we believed that our approach was appropriate given that these documents might partly reflect the research focus in each country.

The thematic maps of authors’ keywords and terms reveal that major research themes included virological and molecular analysis of the virus; clinical, laboratory and radiology examinations; and global and public health responses. Our findings are in line with a previous bibliometric study, which showed that virology, clinical characteristics, and epidemiology of COVID-19 were found to be the major research foci with the highest volume of papers [22]. Indeed, it has been a short period of time since the onset of the pandemic, and these research areas are essential components for preventing and controlling the pandemic. Understanding the biology of SARS-CoV-2 is critical for the development of effective and safe screening tests, drugs, and vaccines, while investigations into clinical and paraclinical characteristics of COVID-19 could inform a fundamental method for appropriate patient management. Research on public health responses could illustrate the effectiveness of different policies and strategies to mitigate the consequences of the COVID-19 pandemic [32,33,34]. Notably, we believed that much research is ongoing as well as numerous papers are under reviewed, which will remarkably contribute to the global knowledge about COVID-19 in the short coming.

Results of topic modeling offer more penetrating insights into the emerging research themes. Of all identified topics, clinical aspects, particularly guidelines for emergency care and surgical management during the COVID-19 pandemic, were most frequent. Along with the rapid increase in the number of confirmed cases, the heavy demand for health facilities and health workers, along with the lack of effective treatment regimens, place a heavy burden and prevent the healthcare systems from operating efficiently. Without guidelines for prompt responses in emergency care, the burden caused by COVID-19 would go beyond the capacity of most health systems, especially for ICU care [35]. In addition, a number of SARS-CoV-2 infections emerged from operations were reported in China, suggesting the risk of virus exposure despite strict hygienic requirements and aseptic techniques during the surgical process [36]. Research for clinical guidelines, therefore, plays a critical role in mitigating the impact of COVID-19 on the healthcare system. 

The origin and pathophysiology of the virus have attracted a great deal of attention since the beginning of the outbreak [37,38,39]. The interest in this topic has continued to rise as the virus has gone beyond China, where the first infection was reported, and positive cases have been found in most countries and territories [40]. On the other hand, the information that SARS-CoV-2 is a laboratory derived virus, albeit that this has been confirmed to be a false claim, gave rise to considerable controversy and also facilitated research on the nature of the virus [41]. Another topic that should be mentioned is national public health responses and actions against COVID-19, especially at the beginning of the pandemic when there was a wide difference in policies introduced by different governments. In particular, some countries advocated achieving herd immunity, whereas low- and middle-income countries (LMICs) implemented strict actions, including quarantine, isolation, social distancing, and community containment as soon as the outbreak occurred [42,43,44,45]. Although such measures have demonstrated their effectiveness, for optimal public health as well as economic outcomes, further investigations into their implementation within specific contextual factors should be prioritized [46]. Moreover, continued medical training for healthcare workers [47] and preventive measures for the workforce [48], along with frequent transparent communication and educational interventions for the public, is essential to strengthen the preventive capacity of each individual and thus, contribute to the global fight against COVID-19. Meanwhile, since COVID-19 has been reported to have no noticeable effect on pregnancy, research on COVID-19 among pregnant women received relatively slight interest [49]. 

Regarding the research foci in different country groups, it appeared that the share of publications regarding psychological health and related interventions was negatively associated with income level. This finding might imply that this topic might not be the priority of the countries, or in other words, developed nations show even less interest than the ones having lower-income [50]. However, COVID-19 caused a significant psychiatric impact [51], and this impact was maintained when the total number of COVID-19 cases continued to rise [52]. Developed nations are not immune from mental health issues and mental health services have often been disrupted during the COVID-19 pandemic [53]. Another reason which might play a role in this phenomenon is that most of the studies about this topic were cross-sectional surveys in the community, which were more affordable for low-income countries to perform compared to other topics. Therefore, the share of publications in this topic in low-income countries might be higher than that in high-income countries. 

In terms of treatment interventions, although all countries are making efforts to develop effective treatment regimens, high-income countries, with their vast financial resources, greater expertise, and infrastructure, demonstrated their bold attempt in this research area [54,55]. Meanwhile, compared to low-income nations, we observed a lower share of SARS-CoV-2 test-related publications among low-middle income countries, which might imply that these countries prioritized to other research fields such as treatment interventions given their resource-constraint [56]. In addition, while rapid transmission of COVID-19 has been triggering a strong need for the development of an effective vaccine, our results show minimal research on this topic. However, we do believe that the amount of research on vaccine development is possibly abundant according to the number of studies about COVID-19 vaccination registered in clinicaltrials.gov and the WHO Trial Registry Network. Because it requires remarkable amount of time to obtain results, the small amount of publications compared with other topics is understandable. 

Findings also suggested that research on comorbidities associated with COVID-19 is relatively underdeveloped in countries with sporadic cases, in contrast with the extensive understanding and research on the effects of comorbidities on COVID-19 in those countries with a high number of infections [57,58]. On the other hand, the increase of transmission level was negatively correlated with the interest in treatment interventions. Although some high-income countries such as the United States, Canada, or the United Kingdom were classified as “community transmission” and greatly contributed to the progress of finding treatment interventions, most of the nations in this category were low-middle income countries (e.g., South American and African countries) and the governments tends to focus on preventive methods to prevent the pandemic from getting worse [2]. 

This study has several implications. To begin with, since there has been anecdotal evidence that promising drugs for COVID-19 such as Lopinavir/ritonavir (LPV/RTV), Chloroquine (CQ), and hydroxychloroquine (H0) have shown no significant benefits to health outcomes of patients, developing effective and safe medications specific for the treatment of COVID-19 is of utmost importance [59,60,61]. Furthermore, the findings show a lack of behavioral psychosocial research on how people react with COVID-19 emergency [32]. Future research should consider the risk factors of psychosocial distress at interpersonal or cultural aspect, impact of mass media and social media on behaviors of population to COVID-19, as well as behavior-change interventions to each research subject. Additionally, we found a lack of research on the social stigma caused by COVID-19. Due to the rapid contagion of the virus, fear and anxiety about being infected can give rise to stigma and discrimination toward people, places, or things. For instance, people associated with the disease, such as being in a neighborhood of high risk or being a civilian of a nation with a high rate of COVID-19 infection, are often stigmatized [62,63]. Stigma can also arise when people are released from quarantine, even though they have been confirmed to be negative and are no longer risk. Although there have been several published guidelines for reducing social stigma related to COVID-19, further investigations into the detrimental effects of social stigma and development of interventions for this problem should be considered [62,63]. Finally, due to the rapid spread of this disease, the vulnerable population, such as the elderly living in nursing homes, workers in industrial zones, refugees, migrants, or persons with disabilities are at higher risk of getting an infection, will need extra precautions [64]. However, these high-risk clusters have not received enough concern from the researchers, even on commentary or local government. More research and preventive actions should be done so as to not leave these people behind [64]. 

To our knowledge, this is the first analysis using text mining and text modeling to investigate the research foci of the worldwide COVID-19 publications. However, some limitations should be noted. The restriction of the search strategy to the English language might not reflect globalized practices and the research priority of a country. Analyses of keywords, titles, and abstracts may not fully reflect the content of articles. However, with the combination of three large datasets and various techniques of text mining, this study is useful for an overview of the research direction. Moreover, our correlation analysis was based on population data, which might not reflect the causes of the research tendency in each country. Since the publications on COVID-19 will rapidly grow in the coming time, further studies should be performed with more advanced techniques to elucidate our findings.

## 5. Conclusions

This study showed that COVID-19 related publications were primarily contributed by major research hubs such as the United States, China, and European countries. Global researchers have been currently focused on clinical management, viral pathogenesis, and public health responses in combating against COVID-19. Meanwhile, little attention has been paid to psycho-social problems or of the impacts of COVID-19 on different vulnerable populations. Findings of this study suggest the need for global research collaboration among high- and low/middle-income countries in the different stages of the pandemic prevention and control. This paper can serve as a reference for governments and research institutions to identify the research priority in their settings and allocate appropriate resources for research on COVID-19.

## Figures and Tables

**Table 1:** Top ten most cited papers.

No.	Title	Journal (IF)	Number of Citations	Main Findings
01	Epidemiological and clinical characteristics of 99 cases of 2019 novel coronavirus pneumonia in Wuhan, China: a descriptive study	The Lancet (IF = 59·1)	319	SARS-CoV-2 infection was of clustering onset, and more likely to affect older males with comorbidities. Patients had clinical manifestations of fever, cough, shortness of breath, muscle ache, confusion, headache, sore throat, rhinorrhea, chest pain, diarrhea, and nausea and vomiting. Imaging examination revealed bilateral pneumonia, multiple mottling, and ground-glass opacity.
02	A familial cluster of pneumonia associated with the 2019 novel coronavirus indicating person-to-person transmission: a study of a family cluster	The Lancet (IF = 59·1)	245	Results confirmed that SARS-CoV-2 was transmitted through person-to-person contact. Older patients (aged >60 years) had more systemic symptoms, extensive radiological ground-glass lung changes, lymphopenia, thrombocytopenia, and increased C-reactive protein and lactate dehydrogenase levels. Phylogenetic analysis of showed that this is a novel coronavirus, which is closest to the bat severe acute respiratory syndrome (SARS)-related coronaviruses found in Chinese horseshoe bats.
03	Clinical characteristics and intrauterine vertical transmission potential of COVID-19 infection in nine pregnant women: a retrospective review of medical records	The Lancet (IF = 59·1)	75	Clinical characteristics of COVID-19 pneumonia in pregnant women were similar to those reported for non-pregnant adult patients. Fevers, cough, myalgia, sore throat, and malaise were also observed. No neonatal asphyxia was observed in newborn babies.
04	Detection of 2019 novel coronavirus (2019-nCoV) by real-time RT-PCR	Eurosurveillance (IF = 7·4)	74	The laboratory diagnostic workflow for detection of SARS-CoV-2 was described and validated.
05	Emerging coronaviruses: Genome structure, replication, and pathogenesis	Journal of Medical Virology (IF = 2·0)	51	Available understanding on genome structure and replication, and functions proteins in coronaviral replication of coronaviruses (CoVs) were reviewed. SARS-CoV-2 has a typical genome structure of CoV and belongs to the cluster of betacoronaviruses, including Bat-SARS-like (SL)-ZC45, Bat-SL ZXC21, SARS-CoV, and MERS-CoV.
06	CT imaging features of 2019 novel coronavirus (2019-NCoV)	Radiology (IF = 7·6)	51	Typical CT findings included bilateral pulmonary parenchymal ground-glass and consolidative pulmonary opacities, sometimes with a rounded morphology and a peripheral lung distribution. Lung cavitation, discrete pulmonary nodules, pleural effusions, and lymphadenopathy were absent.
07	Presumed Asymptomatic Carrier Transmission of COVID-19	JAMA - Journal of the American Medical Association (IF = 51·3)	49	All symptomatic patients had multifocal ground-glass opacities on chest CT, and 1 also had subsegmental areas of consolidation and fibrosis. All the symptomatic patients had increased C-reactive protein levels and reduced lymphocyte counts. The coronavirus may have been transmitted by the asymptomatic carrier.
08	Breakthrough: Chloroquine phosphate has shown apparent efficacy in treatment of COVID-19 associated pneumonia in clinical studies	BioScience Trends (IF = 1·7)	48	Chloroquine phosphate is superior to the control treatment in inhibiting the exacerbation of pneumonia, improving lung imaging findings, promoting a virus-negative conversion, and shortening the disease course according to the news briefing. Severe adverse reactions to chloroquine phosphate were not noted in the patients in trial.
09	Genomic characterization of the 2019 novel human-pathogenic coronavirus isolated from a patient with atypical pneumonia after visiting Wuhan	Emerging Microbes and Infections (IF = 6·2)	46	Genome of SARS-CoV-2 has 89% nucleotide identity with bat SARS-like-CoVZXC21 and 82% with that of human SARS-CoV. Phylogenetic trees of their orf1a/b, Spike, Envelope, Membrane and Nucleoprotein clustered closely with those of the bat, civet, and human SARS coronaviruses.
10	Incubation period of 2019 novel coronavirus (2019- nCoV) infections among travelers from Wuhan, China, 20–28 January 2020	Eurosurveillance (IF = 7·4)	30	The mean incubation period was estimated to be 6.4 days (95% credible interval: 5.6–7.7), ranging from 2.1 to 11.1 days (2.5th to 97.5th percentile).

**Table 2:** Fifteen topics about COVID-19 according to topic modeling.

Topic	Content	Top Ten Most Frequent Terms	*n*	%
Topic 1	Epidemiological reports on COVID-19 outbreaks in different countries.	Covid-19; cases; transmission; first; disease; coronavirus; march; countries; confirmed; and health.	295	5.1
Topic 2	Global and international health security and responses in COVID-19 pandemic crisis.	Health; covid-19; pandemic; public; global; response; community; world; emergency; and outbreak.	571	9.9
Topic 3	SARS-CoV-2 virus structure and molecular analysis.	Sars-cov-2; 2019-ncov; human; coronavirus; sars-cov; protein; virus; viral; spike; and receptor.	231	4.0
Topic 4	Distinguishes between old and novel coronavirus: origin, pathology, and pathogenesis.	Coronavirus; respiratory; novel; china; disease; sars-cov-2; severe; syndrome; acute; and outbreak.	611	10.6
Topic 5	Radiographic detection of COVID-19.	Covid-19; patients; symptoms; pneumonia; chest; clinical; disease; children; findings; and imaging.	310	5.4
Topic 6	Psychological disorders in COVID-19 epidemic: epidemiological characteristics and interventions.	Covid-19; health; mental; during; outbreak; study; social; psychological; anxiety; and media.	256	4.4
Topic 7	Clinical and laboratory examinations in hospitalized patients with COVID-19.	Patients; covid-19; clinical; severe; study; group; cases; disease; results; Wuhan	232	4.0
Topic 8	Comorbidities in patients with COVID-19.	covid-19; patients; disease; respiratory; severe; acute; infection; syndrome; article; and coronavirus.	474	8.2
Topic 9	Impacts of COVID-19 on pregnancy outcomes.	Covid-19; review; studies; evidence; women; pregnant; clinical; research; literature; and results.	156	2.7
Topic 10	Diagnostic values of SARS-CoV-2 tests and improvement strategies.	Sars-cov-2; positive; covid-19; viral; testing; detection; rt-pcr; samples; results; and negative.	234	4.1
Topic 11	Guidelines for emergency care and surgical management during COVID-19 pandemic.	Covid-19; pandemic; patients; during; management; cancer; hospital; recommendations; clinical; and surgery.	669	11.6
Topic 12	Global logistics concerns in COVID-19 prevention, treatment and care.	Covid-19; protective; transmission; healthcare; workers; equipment; personal; during; infection; and staff.	242	4.2
Topic 13	Medical education in COVID-19 pandemic.	Covid-19; pandemic; medical; education; medicine; during; response; lessons; students; and nursing.	561	9.7
Topic 14	COVID-19 epidemiological modelling and forecasting.	Covid-19; cases; china; epidemic; number; outbreak; model; Wuhan; measures; and confirmed.	292	5.1
Topic 15	Treatment interventions against COVID-19.	Covid-19; treatment; drugs; clinical; therapeutic; against; antiviral; sars-cov-2; therapy; and effective.	311	5.4

**Table 3:** Regression models to identify the research trend among countries with different income level and epidemic characteristics.

Topic	World Bank Income Classification ^1^			WHO COVID-19 Transmission Classification ^2^		
	Low-Middle Income Countries	High-Middle Income Countries	High Income Countries	Sporadic Cases	Clusters of Cases	Community Transmission
	Coef. (95%CI)	Coef. (95%CI)	Coef. (95%CI)	Coef. (95%CI)	Coef. (95%CI)	Coef. (95%CI)
Topic 1	4.7 (−4.7; 14.0)	6.8 (−6.9; 20.4)	14 (−6.4; 34.5)	−1.1 (−9.1; 6.9)	3 (−3.8; 9.7)	6.7 (−0.7; 14.1)
Topic 2	3.4 (−12.2; 18.9)	−7.1 (−29.8; 15.5)	−10.4 (−44.4; 23.6)	−5.3 (−18.6; 7.9)	−3 (−14.2; 8.3)	2.2 (−10.1; 14.4)
Topic 3	1 (−2.7; 4.7)	1.9 (−3.5; 7.3)	1.3 (−6.8; 9.4)	−0.9 (−4.1; 2.2)	1 (−1.7; 3.6)	0.2 (−2.7; 3.2)
Topic 4	2 (−9.0; 13.0)	3 (−13.1; 19.0)	8.2 (−15.8; 32.3)	1.2 (−8.2; 10.5)	6 (−2.0; 14.0)	5.2 (−3.4; 13.9)
Topic 5	−1 (−2.7; 0.8)	−0.8 (−3.4; 1.7)	−2.6 (−6.4; 1.2)	−1.5 (−2.9; 0.0)	−1 (−2.3; 0.3)	−1.4 (−2.8; 0.0)
Topic 6	−6.4 (−12.4; −0.4) *	−16.9 (−25.6; −8.1) *	−23.5 (−36.6; −10.4) *	4.6 (−0.5; 9.7)	1.3 (−3.0; 5.7)	2 (−2.7; 6.7)
Topic 7	−0.1 (−1.6; 1.3)	0.3 (−1.8; 2.4)	0.7 (−2.5; 3.8)	0 (−1.2; 1.2)	0.4 (−0.7; 1.4)	0.7 (−0.4; 1.9)
Topic 8	2.8 (−3.4; 9.1)	−0.2 (−9.3; 8.9)	3.4 (−10.3; 17.1)	−5.8 (−11.2; −0.5) *	−4.1 (−8.7; 0.4)	1.7 (−3.3; 6.6)
Topic 9	2.2 (−2.9; 7.2)	0.9 (−6.5; 8.3)	0.9 (−10.1; 12.0)	−0.3 (−4.6; 4.0)	2.1 (−1.6; 5.8)	2.5 (−1.5; 6.4)
Topic 10	−1.5 (−2.9; −0.1) *	−1.9 (−3.9; 0.1)	−2.7 (−5.6; 0.3)	−0.3 (−1.5; 0.8)	0.4 (−0.6; 1.4)	0 (−1.1; 1.0)
Topic 11	−10.2 (−20.9; 0.5)	−13.7 (−29.3; 1.8)	−19.6 (−42.9; 3.8)	−2.2 (−11.3; 6.9)	−0.2 (−7.9; 7.6)	1.7 (−6.8; 10.1)
Topic 12	−1.5 (−4.3; 1.4)	−3.2 (−7.3; 1.0)	−3.1 (−9.2; 3.1)	−0.6 (−3.0; 1.8)	−1.1 (−3.1; 1.0)	−0.8 (−3.0; 1.4)
Topic 13	−4.9 (−13.2; 3.3)	−7.6 (−19.6; 4.4)	−4.4 (−22.4; 13.7)	0.8 (−6.2; 7.8)	2.9 (−3.1; 8.9)	2.1 (−4.4; 8.6)
Topic 14	1.7 (−7.4; 10.8)	−2.5 (−15.7; 10.8)	−3 (−22.8; 16.9)	−1 (−8.8; 6.7)	−2.9 (−9.5; 3.7)	−1 (−8.2; 6.1)
Topic 15	16.6 (6.5; 26.7) *	19.9 (5.2; 34.6) *	34.8 (12.8; 56.8) *	−20.8 (−29.3; −12.2) *	−18.9 (−26.2; −11.6) *	−17 (−24.9; −9) *

* *p* < 0.05; ^1^ Compared to Low-income countries. The model was adjusted to natural logarithm of GDP per capita, number of cases, number of deaths, and WHO COVID-19 transmission classification; ^2^ Compared to Pending classification. The model was adjusted to natural logarithm of GDP per capita, number of cases, number of deaths, and World Bank Income Classification.

## Appendix A

**Table A1:** Search query (Web of Science).

No	Search Query	Result
1	TS = (“COVID-19”)	998
2	TS = (“2019 novel coronavirus”)	151
3	TS = (“2019-nCoV”)	255
4	TS = (“severe acute respiratory syndrome coronavirus 2”)	84
5	TS = (“SARS-CoV-2”)	286
6	#5 OR #4 OR #3 OR #2 OR #1	1304
7	#5 OR #4 OR #3 OR #2 OR #1 Refined by: [excluding] PUBLICATION YEARS: (2011 OR 2003)	1302
8	#5 OR #4 OR #3 OR #2 OR #1 Refined by: [excluding] PUBLICATION YEARS: (2011 OR 2003) AND [excluding] DOCUMENT TYPES: (CORRECTION OR DATA PAPER OR REPRINT )	1281
9	#5 OR #4 OR #3 OR #2 OR #1 Refined by: [excluding] PUBLICATION YEARS: (2011 OR 2003) AND [excluding] DOCUMENT TYPES: (CORRECTION OR DATA PAPER OR REPRINT) AND [excluding] AUTHORS: ( ANONYMOUS)	1247
10	#5 OR #4 OR #3 OR #2 OR #1 Refined by: [excluding] PUBLICATION YEARS: ( 2011 OR 2003 ) AND [excluding] DOCUMENT TYPES: (CORRECTION OR DATA PAPER OR REPRINT ) AND [excluding] AUTHORS: ( ANONYMOUS ) AND [excluding] LANGUAGES: ( GERMAN OR HUNGARIAN OR FRENCH OR PORTUGUESE OR ITALIAN OR SPANISH OR TURKISH OR CZECH OR NORWEGIAN )	1207

**Table A2:** Search query (MEDLINE/PubMed).

No	Search Query	Result
1	Search ((((“COVID-19”[Title/Abstract]) OR “2019 novel coronavirus”[Title/Abstract]) OR “2019-nCoV”[Title/Abstract]) OR “severe acute respiratory syndrome coronavirus 2”[Title/Abstract]) OR “SARS-CoV-2”[Title/Abstract]	5779

**Table A3:** Search query (Scopus).

No	Search Query	Result
1	( TITLE-ABS-KEY ( “COVID-19” ) ) OR ( TITLE-ABS-KEY ( “2019 novel coronavirus” ) ) OR ( TITLE-ABS-KEY ( “2019-nCoV” ) ) OR ( TITLE-ABS-KEY ( “severe acute respiratory syndrome coronavirus 2” ) ) OR ( TITLE-ABS-KEY ( “SARS-CoV-2” ) )	3348
2	( TITLE-ABS-KEY ( “COVID-19” ) ) OR ( TITLE-ABS-KEY ( “2019 novel coronavirus” ) ) OR ( TITLE-ABS-KEY ( “2019-nCoV” ) ) OR ( TITLE-ABS-KEY ( “severe acute respiratory syndrome coronavirus 2” ) ) OR ( TITLE-ABS-KEY ( “SARS-CoV-2” ) ) AND ( EXCLUDE ( PUBYEAR , 2011 ) OR EXCLUDE ( PUBYEAR , 2006 ) OR EXCLUDE ( PUBYEAR , 2004 ) OR EXCLUDE ( PUBYEAR , 2003 ) )	3309
3	( TITLE-ABS-KEY ( “COVID-19” ) ) OR ( TITLE-ABS-KEY ( “2019 novel coronavirus” ) ) OR ( TITLE-ABS-KEY ( “2019-nCoV” ) ) OR ( TITLE-ABS-KEY ( “severe acute respiratory syndrome coronavirus 2” ) ) OR ( TITLE-ABS-KEY ( “SARS-CoV-2” ) ) AND ( EXCLUDE ( PUBYEAR , 2011 ) OR EXCLUDE ( PUBYEAR , 2006 ) OR EXCLUDE ( PUBYEAR , 2004 ) OR EXCLUDE ( PUBYEAR , 2003 ) ) AND ( EXCLUDE ( DOCTYPE , “er” ) OR EXCLUDE ( DOCTYPE , “cp” ) OR EXCLUDE ( DOCTYPE , “dp” ) )	3309
4	( TITLE-ABS-KEY ( “COVID-19” ) ) OR ( TITLE-ABS-KEY ( “2019 novel coronavirus” ) ) OR ( TITLE-ABS-KEY ( “2019-nCoV” ) ) OR ( TITLE-ABS-KEY ( “severe acute respiratory syndrome coronavirus 2” ) ) OR ( TITLE-ABS-KEY ( “SARS-CoV-2” ) ) AND ( EXCLUDE ( PUBYEAR , 2011 ) OR EXCLUDE ( PUBYEAR , 2006 ) OR EXCLUDE ( PUBYEAR , 2004 ) OR EXCLUDE ( PUBYEAR , 2003 ) ) AND ( EXCLUDE ( DOCTYPE , “er” ) OR EXCLUDE ( DOCTYPE , “cp” ) OR EXCLUDE ( DOCTYPE , “dp” ) ) AND ( EXCLUDE ( PREFNAMEAUID , “Undefined#Undefined” ) )	3295
5	( TITLE-ABS-KEY ( “COVID-19” ) ) OR ( TITLE-ABS-KEY ( “2019 novel coronavirus” ) ) OR ( TITLE-ABS-KEY ( “2019-nCoV” ) ) OR ( TITLE-ABS-KEY ( “severe acute respiratory syndrome coronavirus 2” ) ) OR ( TITLE-ABS-KEY ( “SARS-CoV-2” ) ) AND ( EXCLUDE ( PUBYEAR , 2011 ) OR EXCLUDE ( PUBYEAR , 2006 ) OR EXCLUDE ( PUBYEAR , 2004 ) OR EXCLUDE ( PUBYEAR , 2003 ) ) AND ( EXCLUDE ( DOCTYPE , “er” ) OR EXCLUDE ( DOCTYPE , “cp” ) OR EXCLUDE ( DOCTYPE , “dp” ) ) AND ( EXCLUDE ( PREFNAMEAUID , “Undefined#Undefined” ) ) AND ( EXCLUDE ( SUBJAREA , “Undefined” ) ) AND ( EXCLUDE ( LANGUAGE , “Chinese” ) OR EXCLUDE ( LANGUAGE , “German” ) OR EXCLUDE ( LANGUAGE , “Spanish” ) OR EXCLUDE ( LANGUAGE , “French” ) OR EXCLUDE ( LANGUAGE , “Norwegian” ) OR EXCLUDE ( LANGUAGE , “Portuguese” ) OR EXCLUDE ( LANGUAGE , “Italian” ) OR EXCLUDE ( LANGUAGE , “Dutch” ) OR EXCLUDE ( LANGUAGE , “Icelandic” ) OR EXCLUDE ( LANGUAGE , “Korean” ) OR EXCLUDE ( LANGUAGE , “Swedish” ) OR EXCLUDE ( LANGUAGE , “Turkish” ) )	3032
